# Favorable Outcome and Safety of Neoadjuvant Trastuzumab Emtansine (T-DM1) in a HER2-Positive Early Breast Cancer Patient with Severe Renal Disease on Hemodialysis Ineligible for Conventional Chemotherapy: A Case Report

**DOI:** 10.3390/reports6010013

**Published:** 2023-03-02

**Authors:** Suman Kalyan Natarajan, Joice Crystal Danansezhian, Vipulkumar Thummar, Priya Mehta

**Affiliations:** 1 MIOT Institute of Cancer Cure (MICC), MIOT International, Chennai 600089, India; 2 Medical Affairs, Zydus Lifesciences Ltd., Ahmedabad 382481, India

**Keywords:** breast cancer, HER2-positive, T-DM1, hemodialysis, renal disease

## Abstract

Breast cancer is the leading cause of cancer-related death among women worldwide. It is the most common malignancy in middle-age and elderly women already suffering from other comorbidities, such as chronic kidney disease (CKD). Being a heterogeneous disease, it has variable subtype-specific outcomes and responses towards treatment. Patients with human epidermal growth factor receptor 2 (HER2) overexpression are treated with anti-HER2-targeted drugs. With the advent of newer drugs, the usage of HER2 blockade and chemotherapy in the neoadjuvant treatment of HER2-positive early breast cancer management helps to increase the probability of achieving pathological complete response. We herein present a case of a patient with breast cancer with long-standing CKD and on maintenance hemodialysis where treatment with conventional chemotherapy regimens was a concern and managed with an antibody–drug conjugate (ADC), namely T-DM1, in a neoadjuvant setting. The patient showed a favorable outcome, and the tolerance of T-DM1 in this patient was predictable. This is a first-of-its-kind case report, where T-DM1 was used in a neoadjuvant setting for a patient on simultaneous hemodialysis.

## 1. Introduction

Breast cancer (BC) is the most common malignancy in middle-age and elderly women already suffering from other comorbidities, such as chronic kidney disease (CKD). CKD, as such, does not limit patients to adjuvant radiotherapy; however, in the context of systemic treatment, it affects the pharmacokinetics of drugs across a diverse range, increasing their toxicity and the risk of adverse drug responses.

In the context of CKD or HD patients, there are a few unsolved clinical issues, such as the reception of a suboptimal anticancer treatment, dosage adjustment according to pharmacokinetic (PK) and pharmacodynamic (PD) parameters, and the timing of drug administration due to parallel HD sessions.

The clinical management of BC depends on the cancer stage and includes surgery, followed by chemotherapy or radiation therapy, or both as required. CKD does not limit surgery or radiotherapy; however, it affects the PKs of chemotherapeutic drugs to an extent, and might increase their toxicity as well as the risk of adverse drug reactions [1].

Patients with human epidermal growth factor receptor 2 (HER2) overexpression are treated with monoclonal antibodies that target these receptors [2,3]. Currently, HER2-positive breast cancer has shown improved outcomes due to the approval of HER2-targeted treatments in neoadjuvant, adjuvant, and metastatic settings.

Neoadjuvant treatment is now considered an integral part of HER2-positive early breast cancer management. The ultimate aim of neoadjuvant treatment is to prevent disease recurrence, and the use of HER2 blockade as well as traditional chemotherapy drugs remains the standard therapeutic approach to elevate number of patients achieving a pathological complete response.

The usage of HER2 blockade along with chemotherapy helps to increase the probability of achieving pathological complete response [4].

We herein present a case of breast cancer with long-standing CKD and on maintenance hemodialysis where treatment with conventional chemotherapy regimens was a concern and managed with an antibody–drug conjugate (ADC), namely T-DM1, in a neoadjuvant setting.

## 2. Detailed Case Description

In July 2021, a 69 year-old female presented at our tertiary care hospital oncology clinic with a complaint of a lump in the left breast during the past month. The patient had a known case of chronic kidney disease since 2009 and had been on twice-weekly maintenance hemodialysis for four years. She had other comorbidities, namely hypothyroidism and systemic hypertension, which she had had for a decade.

**Figure 1:**
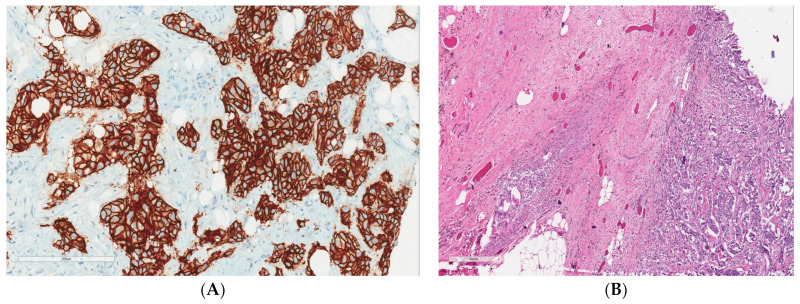
(**A**) Strong membranous positivity of Her2Neu immunohistochemistry with a score of 3 (200×). (**B**) Residual tumor cells and cluster of histiocytes and giant cells (marked by black arrow) upon hematoxylin and eosin staining (200×).

**Figure 2:**
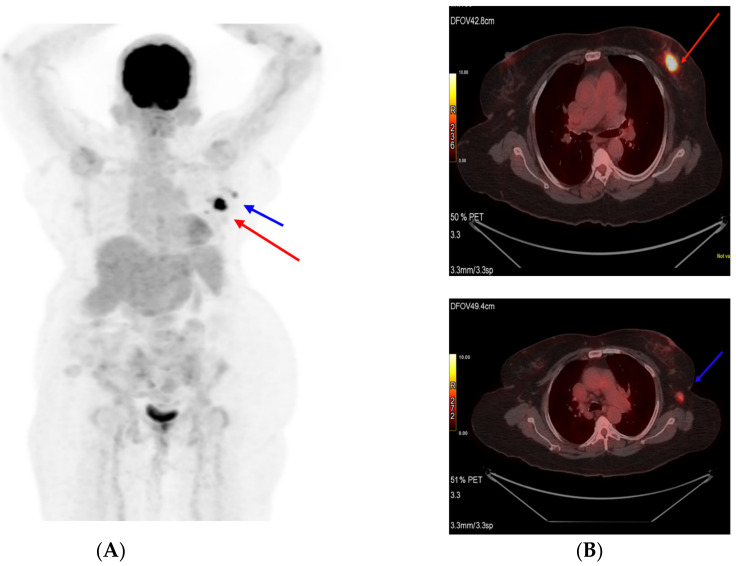
Pretreatment PET scan images (**A**). Primary tumor (Target 1) shown as a red arrow and (**B**) axillary lymph node (Target 2) shown as a blue arrow.

On an initial mammography diagnostic evaluation, a 3 × 2 cm lump was noted in the central and lower outer quadrants of the left breast, which, on further evaluation through ultrasound-guided core needle biopsy, was confirmed as an invasive ductal carcinoma. A pathological examination showed grade II invasive ductal carcinoma of the left breast with axillary lymph nodes with hormone-positive, Her2neu-positive, E-cadherin-positive, and Ki-67 expression of 55 to 60% and of a luminal B type (Figure 1). A whole-body PET CT scan was suggestive of a mass in the lower aspect of the left breast with a few specks of calcifications and left axillary lymphadenopathy (Figure 2). The patient had a previous history of mild anginal symptoms, starting in June 2021, and was evaluated for NSTEMI. The final clinical diagnosis was cT2N1M0-Her2-positive EBC. It was better to proceed with neoadjuvant therapy for three to six cycles and then reassess for surgery.

The patient underwent a full cardiac status evaluation and was found to be eligible for Her2-directed therapy. Due to her CKD status, conventional chemotherapy was not contemplated alongside HER2-targeted therapy. The patient also had concerns related to chemotherapy-related hair loss. Thus, the possible treatment options offered to the patient were dual HER2 blockade (trastuzumab + pertuzumab or trastuzumab + lapatinib) or trastuzumab emtansine (T-DM1). Anthracyclines were never used in this patient due to renal compromise.

**Figure 3:**
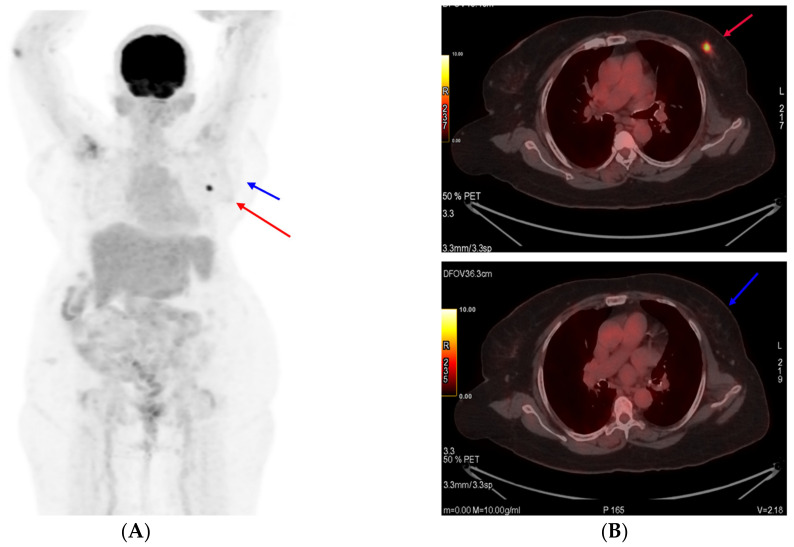
PET scan images at interim assessment after six cycles of T-DM1 (**A**). Primary tumor (target 1) shown as a red arrow and (**B**) axillary lymph node (target 2) shown as a blue arrow.

The patient opted for treatment with T-DM1 after thoroughly understanding the clinical situation and a discussion with the healthcare team. As the patient had access to an approved biosimilar option available in our country, the same was selected and given alongside alternate-day hemodialysis. Thus, the neoadjuvant therapy was started with T-DM1, which the patient tolerated well at a dose of 3.6 mg/kg given once every three weeks. A good partial response was observed after six cycles (Figure 3). During the treatment period, her renal disease parameters also had a correction.

**Figure 4:**
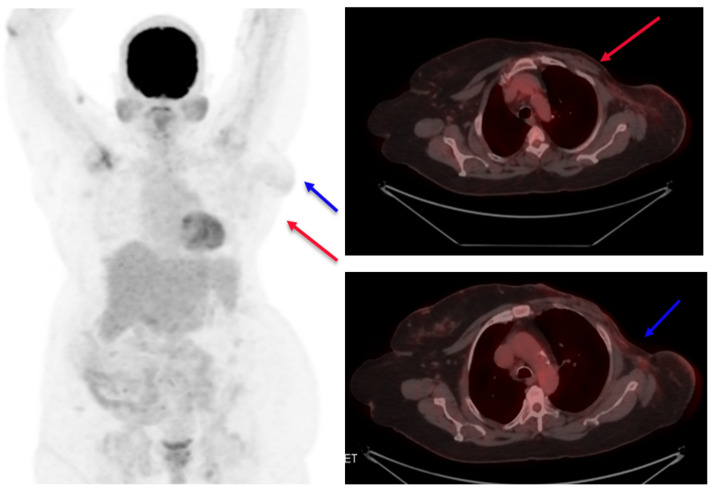
Post-treatment PET scan images. No evidence of the tumor is seen (target 1), shown as a red arrow, and lymph node (target 2) shown as a blue arrow.

A response evaluation via a PET CT scan showed a significant decrease in the size and metabolic activity of the primary left breast lesion, along with regression and metabolic resolution of the left level-I axillary nodes in the patient (partial response, PR, by RECIST 1.1). The patient underwent surgery, namely modified radical mastectomy. A post-operative histopathological examination was suggestive of residual disease with invasive ductal carcinoma, grade: II, tumor size: 1.2 cm, lymph nodes: 1/25, and ypT1cN1aMx. In view of pathological complete response not being achieved, it was decided to continue T-DM1 and complete one year of anti-HER2 therapy. The patient completed one year of T-DM1 therapy with no adverse events. A PET CT scan was performed in May 2022 and showed no evidence of metabolically active disease (Figure 4). She had completed twelve total cycles of T-DM1 and opted to continue with endocrine therapy. She was subsequently initiated on endocrine therapy and continued dialysis. In order to publish this report, written informed consent was obtained from the patient and her legal guardian for the disclosure of images and clinical details.

## 3. Discussion

The advent of newer anti-HER2-targeted drugs, such as pertuzumab and ado-trastuzumab emtansine (T-DM1), has provided benefit to patients, not only in terms of progression-free survival but also in overall survival [5]. The literature is sparse on the usage of these drugs in breast cancer patients with CKD on hemodialysis.

As per the KRISTINE study, in HER2-positive early breast cancer patients the use of neoadjuvant trastuzumab emtansine plus pertuzumab achieved a pathological complete response rate of 44% among subjects. It was a reasonable rate with this regimen, excluding the usage of conventional chemotherapy agents in it, and had a favorable safety profile with a lower incidence of serious and grade ≥ 3 adverse events. In our case, the usage of conventional chemotherapy was a concern because of the renal impairment, while the cost of dual HER2 blockade without chemotherapy had inferior PCR rates compared to an ADC [6]. T-DM1 is an antibody–drug conjugate approved for the treatment of advanced HER2-positive breast cancer. It is also approved for the adjuvant treatment of breast cancer patients who do not achieve complete pathological response after neoadjuvant therapy [5,7].

In the preliminary studies on its pharmacokinetic properties, T-DM1 was found to have almost exclusive hepatic elimination with a minimal presence of T-DM1 metabolites in urine. An exploratory analysis of T-DM1 also states that its pharmacokinetic properties are unaffected by age, race, or renal function [8]. Studies have also proven that patients who with mild or moderate renal impairment, on treatment with T-DM1, exhibited a similar renal profile to patients with normal renal function [9,10].

Thus, in brief, kidney impairment or dialysis should not be considered reasons to limit the usage of or stop an active anti-HER anticancer treatment in patients, especially when there is a possibility of significantly improving the life expectancy of patients with the use of these agents.

T-DM1 is administered i.v. at a dose of 3.6 mg/kg of body weight, every 21 days. Its elimination half-life is approximately 4 days. Due to it having a high molecular weight, T-DM1 clearance is unlikely to impacted by renal function. 

Based on previous preclinical studies, T-DM1 is primarily eliminated through bile after conversion into DM1-containing catabolites, with minimal (\5%) renal elimination. Thus, as per the data, renal impairment is not anticipated to impact T-DM1 pharmacokinetics. Additionally, there are no major data currently available for dialysis patients treated with T-DM1 [11].

We summarized that T-DM1 is found to be an effective and safe drug for HER2-positive breast cancer patients who are on hemodialysis treatment. Though a pharmacokinetic study has not been undertaken here, we found that, for a particular patient, dose adjustment was not necessary as T-DM1 has mainly hepatic clearance and renal excretion is minimal.

The patient reported here showed a favorable outcome, and the tolerance of T-DM1 in this patient was predictable. This is the first case report of T-DM1 in a neoadjuvant setting for a patient on simultaneous hemodialysis. Interestingly, T-DM1 has no reported hair loss, which also helped in the patient’s compliance for one year of treatment.

## 4. Conclusions

T-DM1 is an effective and safe drug for patients with HER2-positive breast cancer who are simultaneously on hemodialysis for CKD. Based on the data of this case report, T-DM1 can be safely administered for EBC patients with comorbidities, such as CKD, and who deserve neoadjuvant Her2-directed therapy.

## Data Availability

Patient data were retrieved from the electronic database of the hospital after obtaining patient consent, and any further queries can be directed toward the corresponding author.

## References

[1] Silvestris N., Argentiero A., Cosmai L., Porta C., Gesualdo L., Brunori G., Brunetti O., Rampino T., Secondino S., Rizzo G. (2019). Management of targeted therapies in cancer patients with chronic kidney disease, or on haemodialysis: An Associazione Italiana di Oncologia Medica (AIOM)/Societa’ Italiana di Nefrologia (SIN) multidisciplinary consensus position paper. Crit. Rev. Oncol. Hematol..

[2] Senkus E., Kyriakides S., Ohno S., Penault-Llorca F., Poortmans P., Rutgers E., Zackrisson S., Cardoso F., ESMO Guidelines Committee (2015). Primary breast cancer: ESMO Clinical Practice Guidelines for diagnosis, treatment and follow-up. Ann. Oncol..

[3] Denduluri N., Chavez-MacGregor M., Telli M.L., Eisen A., Graff S.L., Hassett M.J., Holloway J.N., Hurria A., King T.A., Lyman G.H. (2018). Selection of Optimal Adjuvant Chemotherapy and Targeted Therapy for Early Breast Cancer: ASCO Clinical Practice Guideline Focused Update. J. Clin. Oncol..

[4] Raj S., Franco V.I., Lipshultz S.E. (2014). Anthracycline-Induced Cardiotoxicity: A Review of Pathophysiology, Diagnosis, and Treatment. Curr. Treat. Options Cardiovasc. Med..

[5] Verma S., Miles D., Gianni L., Krop I.E., Welslau M., Baselga J., Pegram M., Oh D.-Y., Diéras V., Guardino E. (2012). Trastuzumab Emtansine for HER2-Positive Advanced Breast Cancer. N. Engl. J. Med..

[6] Hurvitz S.A., Martin M., Symmans W.F., Jung K.H., Huang C.-S., Thompson A.M., Harbeck N., Valero V., Stroyakovskiy D., Wildiers H. (2018). Neoadjuvant trastuzumab, pertuzumab, and chemotherapy versus trastuzumab emtansine plus pertuzumab in patients with HER2-positive breast cancer (KRISTINE): A randomised, open-label, multicentre, phase 3 trial. Lancet Oncol..

[7] Von Minckwitz G., Huang C.-S., Mano M.S., Loibl S., Mamounas E.P., Untch M., Wolmark N., Rastogi P., Schneeweiss A., Redondo A. (2019). Trastuzumab Emtansine for Residual Invasive HER2-Positive Breast Cancer. N. Engl. J. Med..

[8] Lu D., Girish S., Gao Y., Wang B., Yi J.-H., Guardino E., Samant M., Cobleigh M., Rimawi M., Conte P. (2014). Population pharmacokinetics of trastuzumab emtansine (T-DM1), a HER2-targeted antibody–drug conjugate, in patients with HER2-positive metastatic breast cancer: Clinical implications of the effect of covariates. Cancer Chemother. Pharmacol..

[9] Barok M., Joensuu H., Isola J. (2014). Trastuzumab emtansine: Mechanisms of action and drug resistance. Breast Cancer Res..

[10] Sais E., Del Barco S. (2017). A case report of a patient with HER2-Positive metastatic breast cancer on dialysis, who responded to Ado-trastuzumab Emtansine. Ann. Clin. Case Rep..

[11] Cosmai L., Gallieni M., Porta C. (2015). Renal toxicity of anticancer agents targeting HER2 and EGFR. J. Nephrol..

